# Peripheral osteoma of the maxillofacial region: a study of 10 cases

**DOI:** 10.5935/1808-8694.20120006

**Published:** 2015-11-20

**Authors:** Fernando Kendi Horikawa, Ronaldo Rodrigues de Freitas, Fernando Alves Maciel, Antonio José Gonçalves

**Affiliations:** a Maxillofacial traumatology and surgery specialist; Maxillofacial surgeon; b PhD in Medicine - Irmandade da Santa Casa de Misericòrdia de São Paulo; Specialist in Head and Neck and Maxillofacial Surgeries; MD; DDS. Head of the Maxillofacial Surgery Department - Irmandade da Santa Casa de Misericórdia de Sao Paulo); c Maxillofacial traumatology and surgery specialist; Maxillofacial surgeon; d PhD in Medicine - Irmandade da Santa Casa de São Paulo. MD. Head and Neck Surgery Specialist - Department of Surgery - Irmandade da Santa Casa de Misericórdia de Sao Paulo)

**Keywords:** bone neoplasms, jaw neoplasms, neoplasms, osteoma

## Abstract

Peripheral osteoma is a benign neoplasm, with low recurrence rate. Its incidence is rare in the jaws and the mandible is more affected than the maxilla. In most cases it is discovered during routine radiographic examinations.

**Objective:**

The aim of this study is to show the author's experience regarding the treatment of this neoplasm.

**Methods:**

A retrospective study from January 2002 to December 2007 including ten cases of peipheral osteoma in the maxillofacial region which were treated surgically by removal of the lesion followed by histological confirmation. None of the cases were correlated with Gardner's syndrome.

**Results:**

In this series the incidence of this neoplasm was higher in females (1.5:1) with a mean age of 39, without age preference. One of the patients had lesion recurrence two years after the first surgery, having been submitted to another intervention, with no signs of relapse after three years and six months of follow-up.

**Conclusion:**

Surgical treatment is effective for peripheral osteoma with a low recurrence rate.

## INTRODUCTION

Osteoma is a benign osteogenic lesion, characterized by the proliferation of mature cancellous or compact bone^1^^,^^2^.

The osteoma pathogenesis is unknown. Some authors consider it a true neoplasia, while others consider it a hamartoma^3^. Reactional mechanism, trauma or infection are also suggested as possible causes^1^. According to Thoma & Goldman^4^, growth starts spontaneously and is associated to the trauma and not to the inflammation. Schneider et al.^5^ reported on six cases with a positive history of prior trauma. Osteomas a usually located in muscle insertions, suggesting that the muscle pull acts on the development of the lesion. It is very possible that minor traumas, which are not even remembered by the patients, may have caused a subperiosteal hematoma which, associated with the muscle pull, starts the lesion^1^^,^^6^^,^^7^. Varboncoeur et al.^8^ considered the osteoma as cartilage or periosteal embryonic remains.

These lesions are usually small and asymptomatic, usually spotted as radiographic findings, or upon tissue expansion, causing facial asymmetry or functional disorder^8^^,^^9^. Although they may be found at any age, these tumors are more common in young adults, and there is no gender predilection^1^^,^^5^.

Multiple maxilla osteomas associated to other disorders are characteristics found in the Gardner Syndrome^7^^,^^10^, ^11^, ^12^, while single osteomas of the maxillofacial region are considered rare^7^^,^^11^.

Peripheral osteomas of the craniofacial region happen more frequently in the paranasal sinuses. Other locations include the external auditory canal, the orbit, the temporal bone and the pterygoid processes^7^^,^^13^^,^^14^. It is a rare entity in the maxilla and, when the maxillary sinuses are excluded, the mandible is more affected than the maxilla; and the mandibular body and angle are the most commonly affected^7^^,^^11^^,^^14^, ^15^, ^16^.

The traditional radiographic image is usually enough to diagnose an osteoma. It is presented as a radiopaque mass with density similar to that of a normal bone. The panoramic x-ray, that of Waters or CT scan usually shows the location and the benign nature of the lesion^15^.

Histologically, osteomas have two distinct variants. One is made up of relatively dense compact bone with scarce medullary tissue, while the other has lamellar or cancellous bone trabeculae with abundant medullary spaces of fibrous-adipose tissue. Osteoblastic activity is usually prominent^13^^,^^17^.

Osteoma treatment is based on complete surgical removal, on the base, where the bone cortical is located. There are no reports of osteoma malignant transformation^1^^,^^6^^,^^17^.

Osteomas are believed to be relatively uncommon^3^. Its recurrence is rare^8^^,^^18^, with only one case described in the literature^19^.

The goal of the present study was to carry out a retrospective investigation of single peripheral osteomas located in the maxillofacial region, treated in our institution.

## METHOD

From January of 2002 through December of 2007, 10 patients with peripheral osteomas were operated in the Maxillofacial surgery ward of the Surgery Department of the Medical Sciences School of the Santa Casa de São Paulo. Upon reviewing the charts, the following items were assessed: gender, age, location, symptoms, functional involvement, aesthetic involvement and recurrence.

Inclusion criteria were: peripheral osteomas of the maxillofacial region with clinical, image and histopathology diagnosis, with full charts and followed for a minimum period of 12 months.

Exclusion criteria were: peripheral osteomas associated with Gardner's syndrome.

The study was approved by the Ethics in Research Committee of the Irmandade da Santa Casa de Misericórdia de São Paulo, under protocol # 295/08, approved on August 28, 2008.

## RESULTS

We assessed the charts from ten patients, six women, with a female/male ratio of 1.5:1. Age varied between 11 and 61 years, with a mean value of 39 years, without predilection for any age range. They all had a past of facial trauma and the time of follow up varied between one and six years.

Table 1 depicts the distribution of the ten patients according to the location of the lesion, patient gender, symptoms, functional and aesthetic involvement and recurrence.

**Table 1:** Maxillofacial peripheral osteomas: location, gender, symptom, aesthetic and recurrence (n = 10).

Location		Patient	Gender		Pain symptoms	Functional involvement	Aesthetic involvement	Recurrence
			M	F				
	Condyle	3	1	2	3	3	3	–
Mandible	Angle	2	–	2	–	–	2	1
	Parasymphysis	2	2	–	–	–	2	–
	Body	1	–	1	–	–	1	–
Zygoma		2	1	1	–	–	2	–

All the patients were submitted to excisional biopsy. In one case, it was necessary to rebuild the temporomandibular joint with costochondral graft.

**Figure 1:**
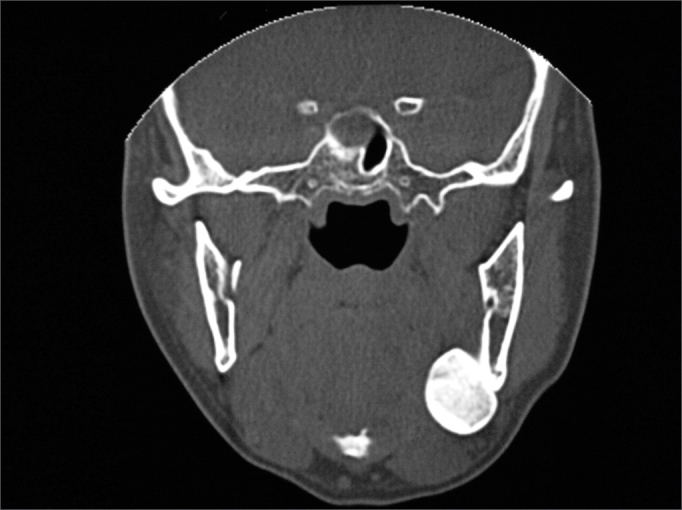
Preoperative aspect: Coronal CT scan showing a peripheral osteoma in the left mandible angle.

**Figure 2:**
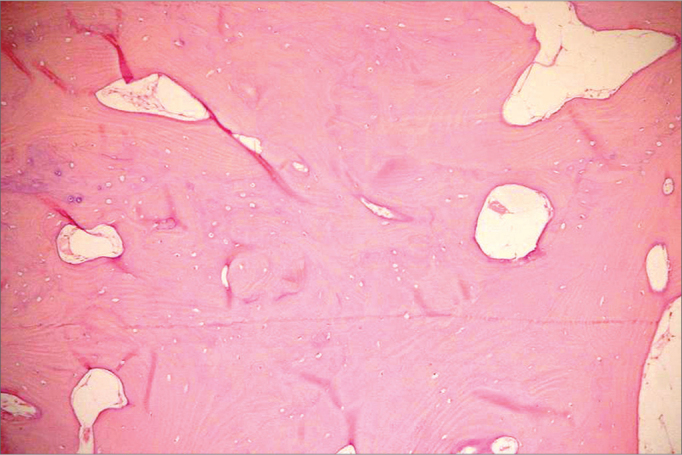
Histological aspect: dense compact bone.

**Figure 3:**
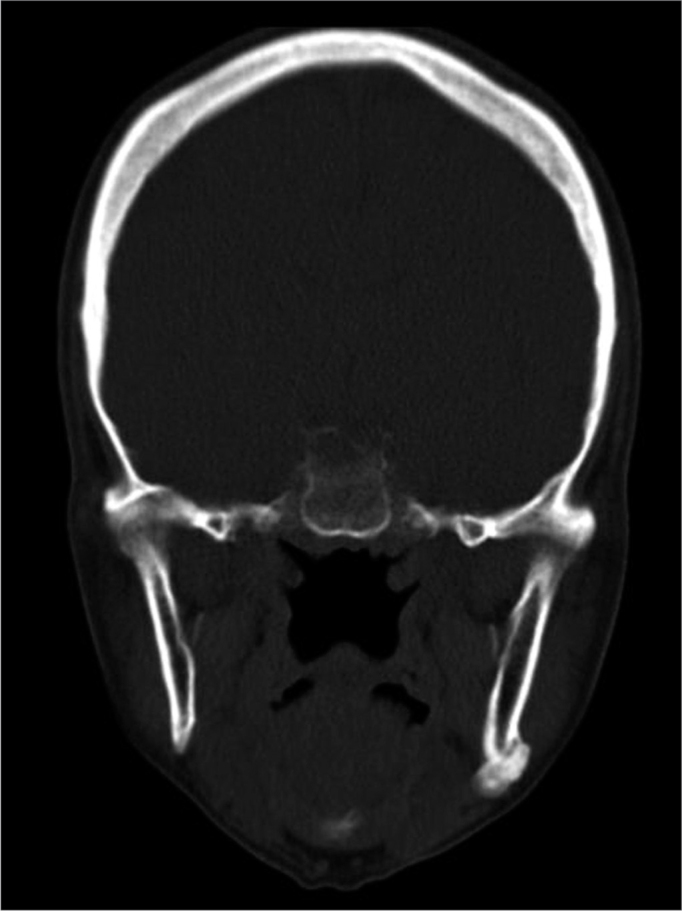
2-year postoperative: Coronal view showing a recurrence in the left mandible angle.

There was one recurrence two years after the surgery (Figure 1, Figure 2, Figure 3). A new surgery was done, and we did not have signs of recurrence after three and a half years of follow up.

**Figure 4:**
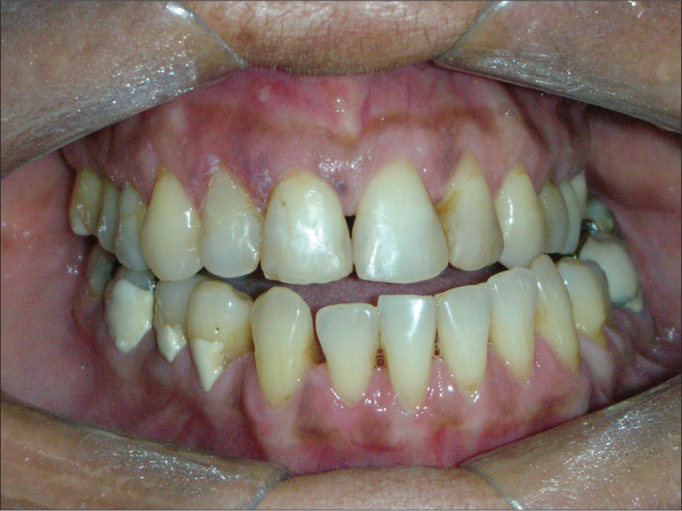
Occlusal changes: deviation from the midline and cross-bite caused by osteoma in the right mandible condyle.

**Figure 5:**
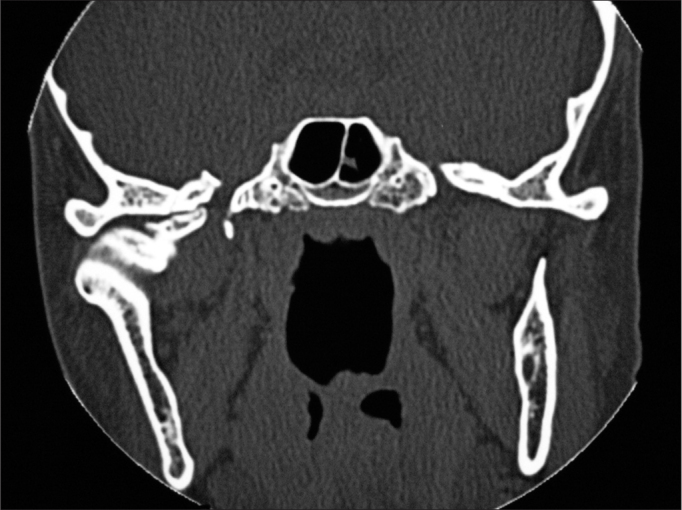
Coronal CT scan: peripheral osteoma in the right-side mandible condyle.

The three cases located in the condyle caused facial asymmetry, dental malocclusion and consequent functional deficit (Figure 4, Figure 5).

## DISCUSSION

The results from this study are in agreement with the opinion given by Ogbureke et al.^2^, that maxillofacial osteomas are frequently detected in routine exams, except in those cases in which the lesions are large enough to cause facial asymmetry or some functional deficit.

According to Bosshardt et al.^19^ and Bessho et al.^20^, peripheral osteomas happen more frequently in the frontal, ethmoid and maxillary sinuses. Nonetheless, Bodner et al.^7^, Sayan et al.^13^ and Johann et al.^14^ state that other topographies in the maxillofacial region may be affected, including the external auditory canal, the orbit, temporal bone, pterygoid process and, rarely, the maxillary sinuses. Schneider et al.^5^ reported 12 cases between 1939 and 1979, and only one happened in the maxilla. Kaplan et al.^6^ added ten other cases between 1985 and 1991.

We agree with the literature in that, when peripheral osteomas affect gnathic bones, they are more frequent in the mandible than in the maxilla. However, just like Bodner et al.^7^, we disagree as to the most affected anatomical region in the mandible, because of the cases studied, most of the lesions were found in the condyle, followed by the angle and parasymphysis, while some of the authors state that the mandible body is the place of the highest incidence^1^^,^^5^^,^^19^.

Cutilli & Quinn^1^ and Bodner et al.^7^ reported that osteomas have no predilection for gender. Notwithstanding, Bosshardt et al.^19^, Kaplan et al.^6^ and Sayan et al.^13^ reported that men are more frequently affected than women, at a 2:1 ratio. Remagen et al.^21^ and Schneider et al.^5^, report an inverted ratio of 3:1. In our study we found a slightly higher prevalence among women, at a 1.5:1 ratio.

According to Bodner et al.^7^, Longo et al.^15^, Sugiyama et al.^16^ and Sayan et al.^13^, there is no predilection for age. Nonetheless, according to Longo et al.^15^, peripheral osteomas are more frequently found in patients between the third and fifth decades of life. Kashima et al.^11^, report that osteomas are more common in the sixth decade of life. The results from this study show that there was no predilection for age, and the lesions were found from the second, all the way to the sixth decade of life.

According to Bosshardt et al.^19^, Bodner et al.^7^, Longo et al.^15^ and Sayan et al.^13^, peripheral osteomas are usually asymptomatic; however, they may be associated with asymmetry or cause malocclusion, impacting the patient's chewing functions. In our study, the patients who had the condyle involved had a shift in their mandibular mid line, cross-bite and reported joint pain, with chewing difficulties.

In image exams, they are usually described as an oval or round mass, limited to a large base. One large single osteoma may look like a parosteal osteogenic sarcoma^15^. Bessho et al.^20^ also include osteochondroma and active hyperplasia of the mandibular condyle in the differential diagnoses. According to Wolford et al.^22^, due to the large similarity of radiographic findings in condyle benign tumors, a conclusive diagnosis can only be established with the microscopic exam.

The CT scan is the best image exam for the diagnosis of peripheral osteomas^7^, because it shows more details about the relationship between the tumor and the adjacent structures, when compared to conventional radiographies^11^. In our cases, the peripheral osteomas were diagnosed by means of routine radiographic exams; nonetheless, the image investigation was complemented by the CT scan, with the goal of enabling a more adequate surgical planning, showing the relationship between the tumor and the adjacent structures, according to Kashima et al.^11^.

Surgery is the treatment of choice; intra or extraoral approaches can be used for the mandible. Intraoral approach is always preferable whenever possible, because it prevents damages to the facial nerve. However, we agree with Longo et al.^15^ who said that in larger tumors located posteriorly on the mandible, the extraoral approach is better, since it provides for a better exposure and visibility - avoiding damage to the important structures in the region. According to these principles, in our services the cases located in the parasymphysis and mandible body we chose the intraoral approach. In the cases located in the mandible angle and condyle, as well as those cases involving the zygoma, we used the extraoral approach.

In those cases involving the mandible, despite the immediate improvement in the post-op and an almost normal mouth opening, the patient will require long term follow up and physical therapy for the masticatory muscles^7^. In this paper, considering all the patients with condyle involvement, we instated forced physical therapy with wood spatulas two weeks after surgery in order to reestablish the mouth opening range seen before surgery.

Recurrence after osteoma surgery is rare^8^^,^^17^^,^^18^; however, Bosshardt et al.^19^ described one case of recurrence nine years after surgical excision. This indicates the need for long standing radiographic and clinical follow up after surgery^13^. Of the ten patients treated in our service, one of them had a recurrence two years after surgery. The patient was submitted to a new procedure and remains without signs of recurrence three year and six months of follow up.

## CONCLUSION

Peripheral osteoma is a rare neoplasia in the maxillofacial region and it more frequently involves the mandible, where the condyle is the preferred site. Females have a higher incidence when compared to males, with no predilection for any specific age range.

Although conventional x-rays provide sufficient information for the diagnosis, today the CT scan is the exam of choice for surgical planning purposes. Surgery with complete lesion removal is the most adequate treatment, with low recurrence rates.
